# A more accurate estimation of the specific surface area of TiO_2_ nanoparticles capped with organic ligands

**DOI:** 10.1039/d5na00732a

**Published:** 2025-08-06

**Authors:** Masahiko Sagawa, Shohei Yamashita, Yohei Okada

**Affiliations:** a Department of Applied Biological Science, Tokyo University of Agriculture and Technology 3-5-8 Saiwai-cho, Fuchu Tokyo 183-8509 Japan yokada@cc.tuat.ac.jp

## Abstract

The specific surface area is important information for nanoparticles. Herein, we demonstrate that treatment with F^−^ ions can strip organic ligands from the surface of TiO_2_ nanoparticles, which enables a more accurate estimation of the specific surface area using N_2_ adsorption–desorption isotherms in conjunction with the Brunauer–Emmett–Teller model.

## Abstract 2

F^−^ ions can strip organic ligands from the surface of TiO_2_ nanoparticles, which enables a more accurate estimation of the specific surface area using N_2_ adsorption–desorption isotherms in conjunction with the Brunauer–Emmett–Teller model.

## Graphical abstract

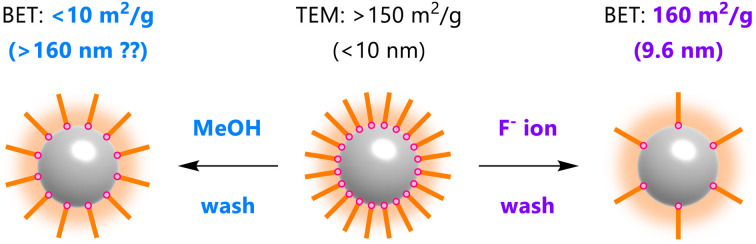

The two decades since the National Nanotechnology Initiative have witnessed a remarkable surge in related research fields.^1^ From the viewpoint of chemistry, producing nanoscale materials that provide a platform to make technology smaller is key. Representative materials are nanoparticles (NPs), also referred as to nanocrystals, and the ability to produce them with controlled size and shape is important. Extensive efforts have established methodologies to realize such controlled production of nanomaterials such as plasmonic NPs (*e.g.*, Au^2–4^ and Ag^5^), quantum dots (*e.g.*, CdSe^6^ and PbSe^7^), magnetic NPs,^8^ and metal oxide NPs (*e.g.*, ZnO,^9^ TiO_2_,^10,11^ and ZrO_2_ ^12,13^), which have found fundamental and practical applications in diverse fields. The successful application of NPs is related to their unique physicochemical properties, particularly their extremely large specific surface areas stemming from their nanoscale dimensions.^14^ Nevertheless, because inorganic and organic components require intrinsically different analysis techniques, quantifying the surface area of NPs remains challenging as a result of the limitations of direct analysis techniques. The difficulty in quantifying surface area is especially true for inorganic NPs capped with organic ligands, which are critical components for both the production and storage of the materials. N_2_ adsorption–desorption isotherms in conjunction with the Brunauer–Emmett–Teller (BET) model are widely used as a conventional approach for estimating the surface area of nanoscale materials, including NPs. However, careful consideration is required when applying the BET method to inorganic NPs capped with organic ligands because the adsorption (desorption) behavior of N_2_ on inorganic materials can differ from that on organic compounds.

Given these analytical limitations, attaining a reasonably accurate estimation of the specific surface area remains one of the most critical challenges in NP characterization, particularly because quantification of ligand density (molecules per nm^2^) is a fundamental component for understanding NP surface chemistry.^15,16^ Although such estimations may be possible for nanospheres through measurement of their diameters,^17^ they are not practical for nanomaterials with different shapes such as nanorods, where large errors would be inevitable.^18,19^ In addition, size and shape distributions, which are generally unavoidable for prepared NPs, complicate the situation because many assumptions are no longer valid. We found a substantial discrepancy between our inorganic NPs' specific surface area determined from BET plots and that expected from TEM observations. This discrepancy has been a persistent challenge in our research, and similar findings have been reported by other researchers.^20^ Although bare pristine particles are preferable for acquiring isotherms, organic ligands are often required in the synthesis stage for size- and shape-controlled NPs,^19,21,22^ where the complete removal of native ligands can be difficult. Although these native ligands can be burned off before the isotherm is recorded, thermal treatment may alter the surface properties.^23^ The removal of such native ligands from the surface of NPs under mild conditions would therefore be helpful. Described herein is an efficient method for removing carboxylic acid and amine ligands from the surface of TiO_2_ nanoparticles with various sizes and shapes, enabling an accurate determination of their specific surface area.

The present work began with the synthesis of TiO_2_ NPs of different sizes and shapes. Using the methodology reported by Do and coworkers,^10^ we synthesized a series of TiO_2_ NPs capped with oleic acid (OA) and/or oleylamine (OAm) using a simple solvothermal route. Do and coworkers found that varying the molar ratio of OA and OAm (defined as *X* = [OA]/([OA] + [OAm])), the amount of titanium(iv) tetrabutoxide used as a precursor, or the reaction temperature enabled fine control of the shape of the TiO_2_ NPs.^10^ Herein, we attempted to prepare TiO_2_ NPs using several different OA/OAm molar ratios, where the amount of titanium(iv) tetrabutoxide and the reaction temperature were fixed (see SI for details). As a result, we obtained TiO_2_ NPs with different shapes capped with OA and/or OAm as hydrophobic precipitates in aqueous ethanol, including spherical-, rhombic-, and rod-shaped NPs (Fig. 1 and S1 in the SI). The precipitates were collected by centrifugation with *n*-hexane and ethanol (1 : 3 v/v), and the excess OA and/or OAm were rinsed away. For further characterization, the precipitates were dispersed in chloroform as a good solvent for the ligand-capped TiO_2_ (refer to the SI for the detailed procedure). All of the dispersions in chloroform were transparent (Fig. 2), suggesting that OA and/or OAm are effective ligands that can prevent agglomeration of the TiO_2_ NPs. Ultraviolet-visible (UV-vis) absorption spectroscopy quantitatively supported the light transmittances when the transmittances were measured at 550 nm. The dispersions were subsequently evaluated by dynamic light scattering (DLS) measurements to ensure that the TiO_2_ NPs were not substantially agglomerated.

**Fig. 1:**
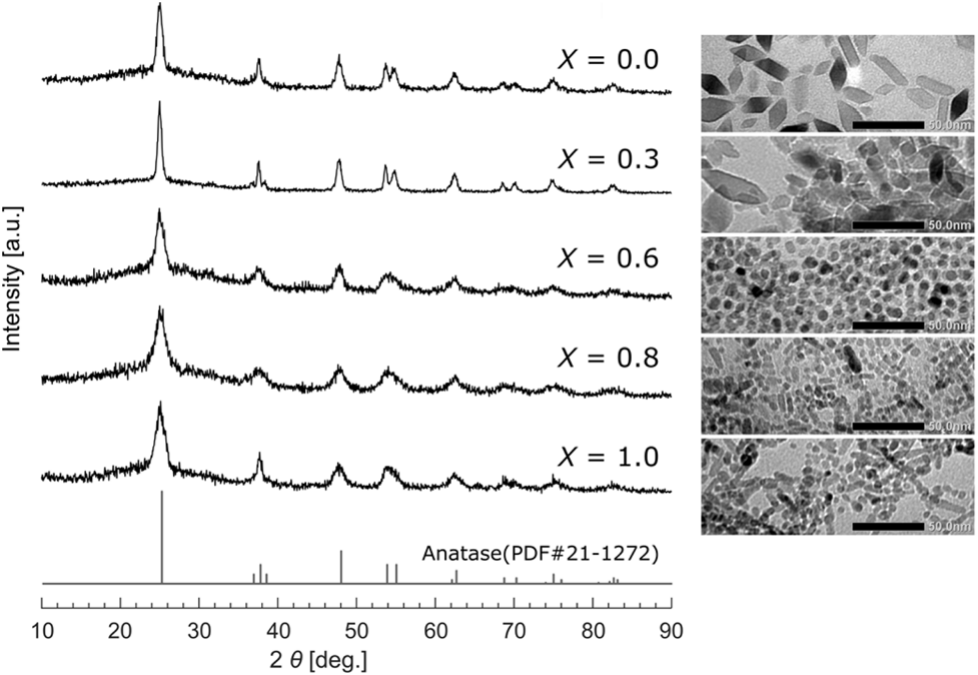
XRD patterns and TEM images of synthesized TiO_2_ NPs. *X* = [OA]/([OA] + [OAm]). The scale bars are 50 nm.

**Fig. 2:**
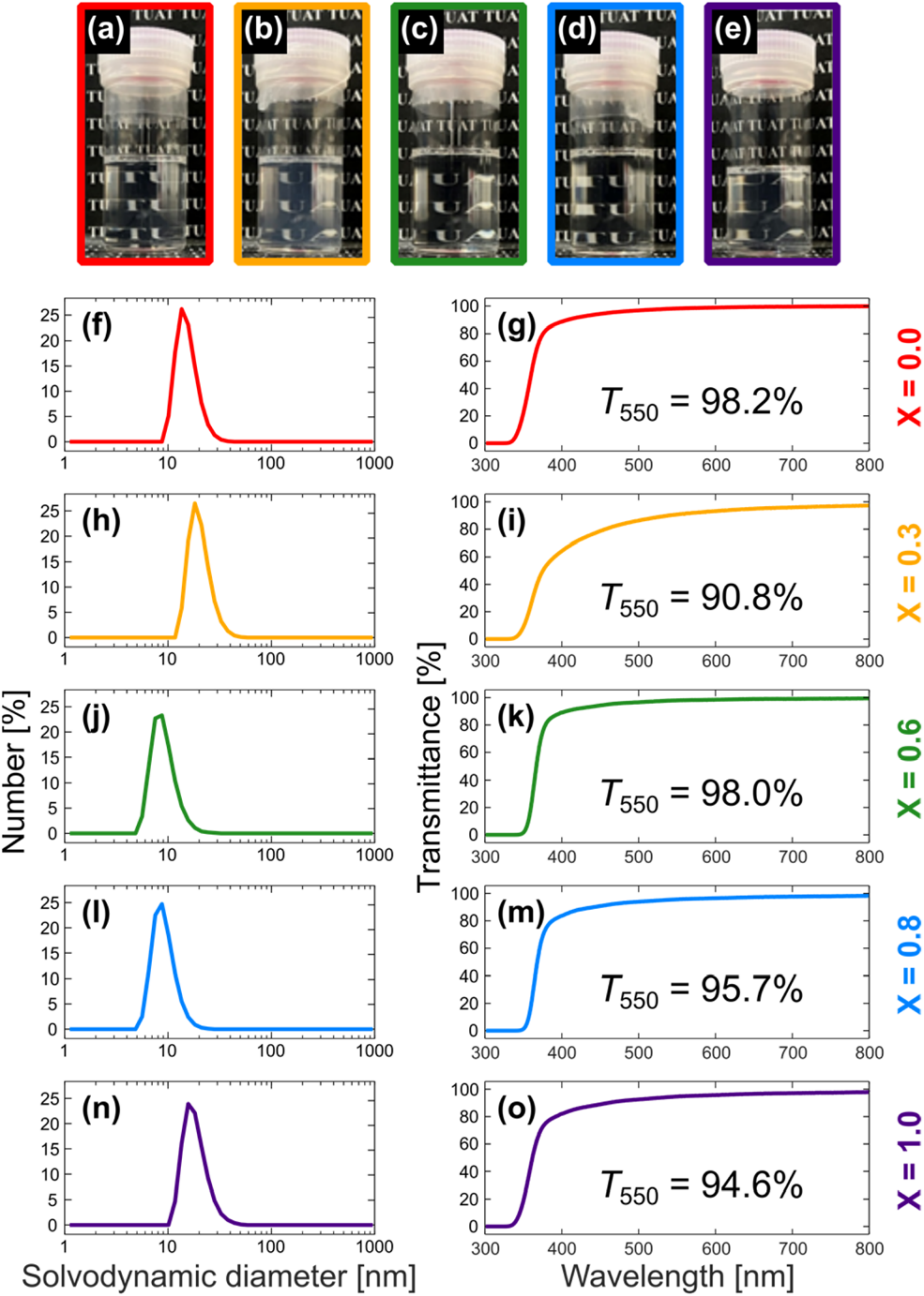
Colloidal characteristics of as-synthesized TiO_2_–OA/OAm in chloroform. (a)–(e) Photos, (f), (h), (j), (l) and (n) solvodynamic size distributions obtained by DLS measurement, and (g), (i), (k), (m) and (o) light transmittance obtained by UV-vis spectroscopy of colloidal solutions of TiO_2_ NPs in chloroform in cases of (a), (f) and (g) *X* = 0.0, (b), (h) and (i) *X* = 0.3, (c), (j) and (k) *X* = 0.6, (d), (l) and (m) *X* = 0.8, and (e), (n) and (o) *X* = 1.0, where *X* = [OA]/([OA] + [OAm]).

Although defining the “diameter” of the TiO_2_ NPs with different sizes and shapes is not straightforward, DLS measurements indicated that the solvodynamic diameters were in the range 8–19 nm. Transmission electron microscopy (TEM) images showed that the TiO_2_ NPs were approximately 5–25 nm in diameter, in accordance with the DLS measurements. The X-ray diffraction (XRD) patterns for all the TiO_2_ NPs indicate single anatase phases, and the crystallite sizes calculated on the basis of the Scherrer formula for the peak at 2*θ* = 25° were estimated to be 5–14 nm. Collectively, these results indicate that the synthesized TiO_2_ NPs were approximately 5–25 nm nanoscale materials (Tables 1 and S1 in the SI).

**Table 1:** Estimated NP size of TiO_2_–OA/OAm, as obtained from different measurements. *X* = [OA]/([OA] + [OAm])

Method	Estimated size [nm]				
	*X* = 0.0	*X* = 0.3	*X* = 0.6	*X* = 0.8	*X* = 1.0
TEM (short)^a^	10.8 ± 2.4	12.5 ± 4.1	6.7 ± 0.8	4.8 ± 1.2	5.3 ± 1.3
TEM (long)^a^	23.6 ± 7.3	24.9 ± 11.2	9.2 ± 2.0	10.4 ± 3.9	12.3 ± 4.9
DLS^b^	14.8	19.6	8.7	8.8	17.6
BET^c^ (MeOH)	38.9	39.1	160.3^d^	1183^d^	26.1
BET^c^ (F^−^ ion)	14.9	17.7	9.6	8.5	9.0

a Values are expressed as mean ± SD. The terms “short” and “long” denote the minor and major axes, respectively, of non-spherical TiO_2_ NPs. TEM images and size quantification results are shown in Fig. S1.

b Values are expressed as median diameters of number-based solvodynamic size distributions shown in Fig. 2.

c Values are calculated under the assumption of truly spherical anatase particles.

d Accuracy is relatively poor because of the low specific surface area obtained using the BET method.

In the case of the specific surface area, however, an accurate estimation remained challenging. Here, the simplest true-spherical variant is considered as a model for a case study. Because the density of TiO_2_ is 3.90 g cm^−3^, the specific surface area of a uniform true sphere of TiO_2_ with a diameter of 8 nm, as a representative size of spherical TiO_2_ NPs (*X* = 0.6), can be calculated to be 192 m^2^ g^−1^. However, in the case of spherical NPs (*X* = 0.6), the specific surface area of the TiO_2_ NPs estimated from the N_2_ adsorption–desorption isotherm using the BET model was found to be 9.6 m^2^ g^−1^, with substantial experimental errors. The actual TiO_2_ NPs were neither uniform nor true spheres; nonetheless, a ∼20-fold difference is not acceptable. Similar results were obtained for other TiO_2_ NPs with different sizes and shapes (Table 2). A thermal treatment at 600 °C to burn off OA and OAm before the isotherm was recorded had some effect; however, the effect was still insufficient because the specific surface area was estimated to be ∼30 m^2^ g^−1^ (Fig. S2 in the SI). Although the mechanism remains an open question, OA and/or OAm clearly affect the N_2_ adsorption (desorption) behaviour of the as-synthesized TiO_2_ NPs.

**Table 2:** Specific surface area of TiO_2_–OA/OAm particles subjected to different wash methods, as measured by N_2_ adsorption–desorption isotherms based on the BET model. The isotherms are shown in Fig. S3 and S4. *X* = [OA]/([OA] + [OAm])

Wash method	Specific surface area [m^2^ g^−1^]				
	*X* = 0.0	*X* = 0.3	*X* = 0.6	*X* = 0.8	*X* = 1.0
MeOH	39.5	39.3	9.6	1.3	58.8
F^−^ ion	103.6	87.0	160.3	181.8	171.8

In this context, Reimhult and coworkers reported a methodology in which F^−^ ions were used to remove OA ligands from the surface of Fe_3_O_4_ NPs with unprecedented efficiency.^24^ Their results showed that the OA ligands capping the surface of Fe_3_O_4_ NPs were fully stripped when the NPs were treated with F^−^ ions. We applied this procedure^24^ to TiO_2_ NPs capped with OA and/or OAm (refer to the Experimental section for details). To quantify the surface coverage, thermogravimetric analysis (TGA, Fig. S5 in the SI) and CHN elemental analysis were performed and the results were compared. In all cases of *X*, TGA showed larger surface coverage than CHN elemental analysis. We speculated that TGA might have overestimated the surface coverage because of residual solvents such as water on the surface of the TiO_2_ NPs. We therefore chose CHN elemental analysis as the method for quantifying the surface coverage, under the assumption that all carbon detected by elemental analysis originated from the surface ligands. A series of CHN elemental analyses showed that 3–11% of the contents of the TiO_2_ NPs were organic even after the samples were washed twice with methanol, which is known to be an effective solvent for removing carboxylate ligands.^25^ We attribute these results to the presence of OA and OAm ligands on the NPs' surface. By contrast, after the treatment with F^−^ ions, the organic content was reduced to <2% (Fig. 3). Except for aggregation due to the removal of surface ligands, the shape and size of the TiO_2_ NPs did not substantially change between before and after the treatment with F^−^ ions or with methanol, as revealed by TEM observation (Fig. S6 in SI). The XRD patterns (Fig. 4) further corroborated these findings: the diffraction patterns for the F^−^-washed TiO_2_ NPs remained identical to those for the methanol-washed samples, preserving the original crystallinity. By contrast, the patterns for the thermally treated samples exhibited slightly sharper peaks, indicating that the thermal treatment process altered the crystalline structure.

**Fig. 3:**
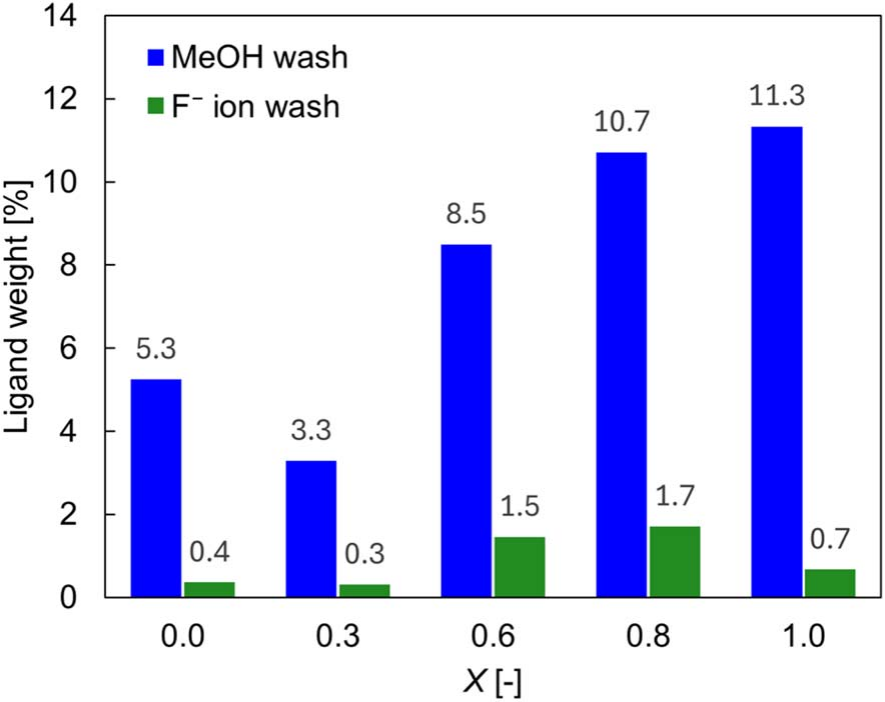
Ligand weight of TiO_2_ NPs washed with (blue) methanol and (green) F^−^ ions, plotted as a function of the OA/OAm ratio (*X* = [OA]/([OA] + [OAm])). The values were obtained by elemental analysis.

**Fig. 4:**
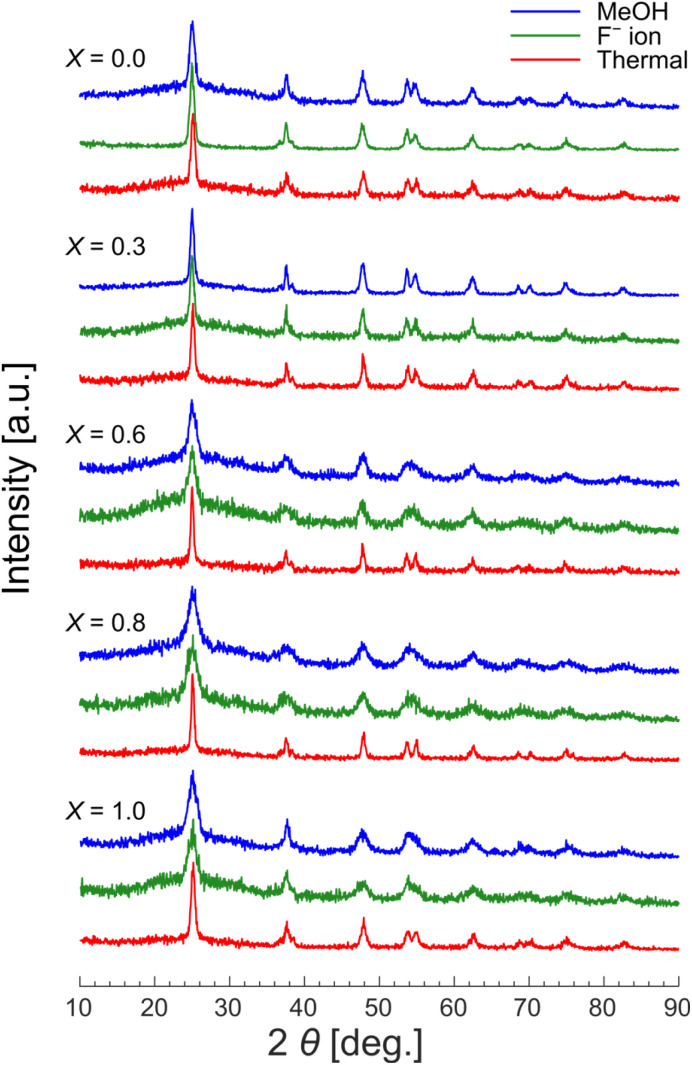
XRD patterns for TiO_2_ NPs for comparison between three strategies of surface ligand removal: (blue line) washing with methanol, (green line) washing with F^−^ ions, and (red line) thermal treatment at 600 °C. *X* = [OA]/([OA] + [OAm]).

To further investigate the removal of the organic ligands, we characterized the NPs by FT-IR spectroscopy (Fig. S7 in the SI). Asymmetric and symmetric CH_2_ stretching vibrations (2920 and 2850 cm^−1^),^26^ a C=O stretching vibration (1640 cm^−1^),^26^ and asymmetric and symmetric COO^−^ stretching vibrations (1520 and 1412 cm^−1^)^27^ were observed and are attributed to residual ligands. The FT-IR analysis revealed the presence of residual organic ligands in all cases of TiO_2_ NPs washed with methanol, in good agreement with the CHN elemental analyses. The aforementioned results demonstrate that the procedure for stripping OA ligands from the surface of Fe_3_O_4_ NPs^24^ can also be used to remove organic capping ligands on TiO_2_ NPs.

Although the removal was not completed, the effect on the N_2_ adsorption–desorption isotherms was evident. The treatment with F^−^ ions to strip OA and/or OAm ligands before acquisition of the isotherm strongly affected the isotherm, and the specific surface area of the TiO_2_ NPs was estimated to be ∼160 m^2^ g^−1^ for *X* = 0.6. This value is comparable to the calculated value of 137–261 m^2^ g^−1^ estimated by TEM observation (Table S2 in the SI for details). Similar results were obtained for other TiO_2_ NPs with different sizes and shapes (Table 2). Because the organic content was measured to be 8.5% for the *X* = 0.6 sample after the treatment with methanol, the surface coverage was estimated to be 1.3 molecules per nm^2^ (see SI for details). Notably, this calculation does not include any assumptions about the particle shape (*e.g.*, true sphere or spheroid). Similarly, for the *X* = 0.0, 0.3, 0.8, and 1.0 samples after the treatment with methanol, the surface coverage was estimated to be 1.2, 0.87, 1.4, and 1.6 molecules per nm^2^, respectively.

## Conclusions

We have demonstrated that the treatment with F^−^ ions can be used to strip organic ligands from the surface of TiO_2_ NPs, which enables a more accurate estimation of the specific surface area using N_2_ adsorption–desorption isotherms in conjunction with the BET model. The methodology described herein is effective for estimating the surface coverages of the TiO_2_ NPs of different sizes and shapes, which we believe is one of the most important pieces of information for inorganic NPs capped with organic ligands. Further studies focusing on the mechanistic aspect of understanding the detailed surface chemistry of inorganic NPs capped with organic ligands are underway in our laboratory.

## Conflicts of interest

There are no conflicts to declare.

## Supplementary Material

NA-007-D5NA00732A-s001

## Data Availability

The data supporting this article have been included as part of the SI. Supplementary information is available: Experimental methods and additional tables and figures. See DOI: https://doi.org/10.1039/d5na00732a.
