# Supplementary material for: A more accurate estimation of the specific surface area of TiO2 nanoparticles capped with organic ligands
Source: Nanoscale Adv. 2025 Aug 6;7(17):5161–5. doi: 10.1039/d5na00732a (PMC12337766; doi:10.1039/d5na00732a)
Supplement: NA-007-D5NA00732A-s001 [file NA-007-D5NA00732A-s001.pdf]

Supplementary Information (SI) for Nanoscale Advances.
This journal is © The Royal Society of Chemistry 2025

Supporting Information for:

A more accurate estimation of the specific surface area of TiO_2 nanoparticles capped with organic ligands.

*Masahiko Sagawa, Shohei Yamashita, and Yohei Okada**

Department of Applied Biological Science, Tokyo University of Agriculture and Technology, 3-5-8 Saiwai-cho, Fuchu, Tokyo 183-8509, Tokyo, Japan.

Table of Contents:

S1. Experimental Methods

S2. Additional Tables and Figures

S3. References

Section S1: Experimental Methods

Materials.

Ethanol (>99.5%, Kanto Chemical Co., Inc.), oleic acid (OA) (>85.0%, Tokyo Chemical Industry Co., Ltd.), oleylamine (OAm) (>50.0%, Tokyo Chemical Industry Co., Ltd.), tetrabutyl orthotitanate (Tokyo Chemical Industry Co., Ltd.), chloroform (>99.0%, Kanto Chemical Co., Inc.), *n*-hexane (>95.0%, Kanto Chemical Co., Inc.), Sodium Fluoride (>99.0%, Tokyo Chemical Industry Co., Ltd), 15-Crown 5-Ether (>97.0%, Tokyo Chemical Industry Co., Ltd), Isopropanol (>99.5%, Kanto Chemical Co., Inc.), methanol (>99.5%, Kanto Chemical Co., Inc.), and acetone (>99.5%, Kanto Chemical Co., Inc.) were used as received without further purification.

Synthesis of TiO_2 NPs.

Synthesis of oleic acid (OA) and oleylamine (OAm)-capped TiO_2 colloidal NPs. Oleic acid- and oleylamine-capped TiO_2 colloidal nanoparticles (NPs) are synthesized according to the method of Do *et al.*[1] The precursor solution was prepared as follows; For the case of the molar ratio of OA and OAm (which is defined as X = [OA]/([OA]+[OAm])) equals to 0.6, to 2.92 mL (50 mmol) of EtOH was added 4.76 mL (15 mmol) of OA and 3.22 mL (10 mmol) of OAm and stirred at room temperature for ca. 5 min until homogeneous solution is formed. Then, 850 μL (2.5 mmol) of tetrabutyl orthotitanate was added and the mixture was stirred at room temperature for ca. 20 min. The obtained precursor solution was placed in a 3 mL vessel placed in a Teflon cup and a mixture of ethanol and water (96% v/v ethanol) was added to the outside of the vessel. The Teflon cup was placed in an autoclave and heated at 180 °C for 18 h at a heating rate of 10 °C/min. The obtained white precipitates were washed once by centrifugation at 12,000 rcf for 5 min with

a mixed solution of *n*-hexane and ethanol (1:3 v/v). The obtained TiO_2 NPs were dispersed in chloroform, and this was used as the stock solution. For the cases of X = 0.0, 0.3, 0.8 and 1.0, to 2.92 mL (50 mmol) of EtOH was added 8.06 mL (25 mmol) of OAm, 2.38 mL (7.5 mmol) of OA and 5.64 mL (17.5 mmol) of OAm, 6.35 mL (20 mmol) of OA and 1.61 mL (5 mmol) of OAm, and 7.93 mL (25 mmol) of OA, respectively.

Wash of TiO_2 NPs.

Crown ether-assisted wash of OA/OAm-capped TiO_2 NPs by F^- ion. The crown ether-assisted wash is carried out according to the literature.[2] A sodium fluoride (NaF)/crown ether aqueous solution is prepared by dissolving 50 mg of NaF in 1 mL of deionized water, followed by the addition of 250 mg of 15-Crown 5-Ether. The mixture was stirred until homogeneous solution was obtained. Subsequently, 7 mL of isopropanol and the NaF/crown ether aqueous solution were added to 10 mL of ligand-capped TiO_2 dispersion in chloroform (equivalent to 250 mg of solid), and the resulting mixture was stirred for 30 min. The solution was transferred to a 40 mL centrifuge tube and diluted to the fill line with isopropanol. After centrifugation at 12,000 rcf for 5 min, the supernatant was discarded. The precipitates were resuspended by adding 10 mL of chloroform, forming turbid solution. The precipitates were collected by addition of methanol, centrifugation at 12,000 rcf for 5 min and decantation, and this centrifugation/decantation step was repeated once. After that, the precipitates were washed twice with deionized water and once with acetone by centrifugation at 12,000 rcf for 5 min each. The resulting precipitates were dried under vacuum overnight at room temperature prior to analyses.

Wash of OA/OAm-capped TiO_2 NPs by methanol. For X = 0.0, 0.3, 0.6, or 0.8; 35 mL of methanol was added to 5 mL of ligand-capped TiO_2 dispersion in chloroform (equivalent to 50–100 mg of solid) in a 40 mL centrifuge tube. After centrifugation at 30,000 rcf for 5 min, the supernatant was discarded. The precipitates were resuspended by adding 5 mL of chloroform. The precipitates were collected by addition of 35 mL of methanol, centrifugation at 30,000 rcf for 5 min and decantation. For X = 1.0; 5 mL of ligand-capped TiO_2 dispersion in chloroform (equivalent to ca. 100 mg of solid) was evaporated and resuspended in methanol, forming turbid solution. The solution was transferred to a 40 mL centrifuge tube and diluted to the fill line with methanol. After centrifugation at 30,000 rcf for 5 min, the supernatant was discarded. The precipitates were resuspended by adding 1 mL of chloroform. The precipitates were collected by addition of 39 mL of methanol, centrifugation at 30,000 rcf for 5 min and decantation.

Thermal treatment of OA/OAm-capped TiO_2 NPs. The TiO_2 NPs washed by methanol as described above were heated to 600 °C at a heating rate of 10 °C/min with no retention time. After cooling to room temperature, the samples were used for analysis.

Calculation of Surface Coverage.

The surface coverage θ was calculated using the following formula;

$$\theta = \frac{w}{(100 - w)} \cdot \frac{N_{\mathrm{A}}}{M_{\mathrm{W}} \cdot SSA}$$

$$w = \frac{carbon\ \%\ in\ sample}{carbon\ \%\ in\ ligand} \times 100\ [\%]$$

where N_A is the Avogadro constant, M_W is the molecular weight of surface ligands, and *SSA* is the specific surface area. The value of carbon % in sample was obtained by CHN elemental analysis.

Characterizations.

X-ray diffraction (XRD) measurements. The crystal structure was examined by XRD using Rigaku RINT-2000 with a Cu Kα source operated at 40 kV and 30 mA.

Transmission electron microscopy (TEM) observation. The size and morphology of synthesized TiO_2 NPs are observed by TEM using JEOL JEM-1400 and JEM-1400Flash.

Dynamic light scattering (DLS) measurements. Zetasizer Nano ZS (Malvern Panalytical Ltd.) with quartz cells were used to measure the solvodynamic diameters. Colloidal solutions were prepared at a concentration of 1 mg of capped NPs per 1 mL of chloroform. All measurements were performed at 20 °C.

Visible light transmittance. All visible light transmittances were measured on a JASCO V-750 spectrophotometer using a quartz cell. The colloidal solutions were prepared at a concentration of 1 mg of capped NPs per 1 mL of solvents.

Thermogravimetry analysis. Removal of surface ligands were quantitatively evaluated by thermal analysis using Rigaku Thermo plus EVO TG8120 and Thermo plus EVO2 TG-DTA8122 under air at a heating rate of 10 °C/min.

Elemental analysis. Removal of surface ligands were quantitatively evaluated by CHN elemental analyses using a PerkinElmer Series II CHNS/O analyzer 2400. All samples were enclosed in Sn sample pans and then introduced into the analyzer. The ligand mass was calculated from the carbon mass percentage detected in the sample with the assumption that all carbon detected by elemental analysis originated from the ligand and, in the case of mixed OA/OAm ligands, that the initial ligand feeding ratio corresponded to the ratio of ligands on the surface.

N_2 adsorption−desorption isotherms. The specific surface areas of NPs are determined based on the Brunauer−Emmett−Teller (BET) theory by N_2 adsorption−desorption isotherms using BEL Japan BELSORP-max.

Attenuated total reflectance-Fourier transform infrared spectroscopy (ATR-FTIR) measurements. ATR-FTIR spectra were measured on a JASCO FT/IR-4100 spectrometer to characterize the surface ligands.

Section S2: Additional Tables and Figures

Table S1. Crystallite sizes (nm) of synthesized TiO_2 NPs, as estimated from the (101) diffraction peak ($2\theta = 25°$) using the Scherrer formula.

X = [OA]/([OA] + [OAm])	Crystallite size [nm]		
	MeOH wash	F^{-}-ion wash	Thermal treatment
0.0	12.9	11.1	11.0
0.3	14.3	13.8	15.6
0.6	6.7	6.7	19.5
0.8	5.1	5.1	18.1
1.0	5.7	6.2	15.4

$X = 0.0$

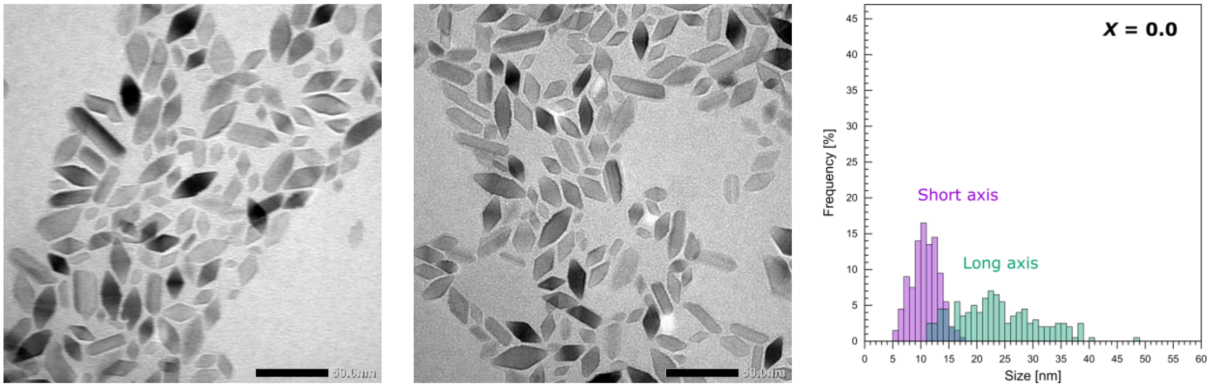

X = 0.0
Short axis
Long axis
Frequency [%]
Size [nm]

$X = 0.3$

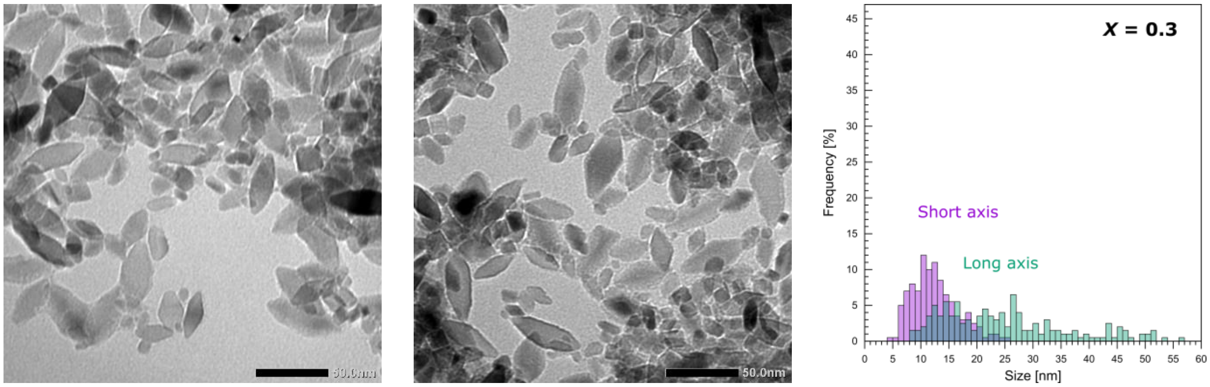

50.0nm
X = 0.3
Short axis
Long axis
Frequency [%]
Size [nm]

$X = 0.6$

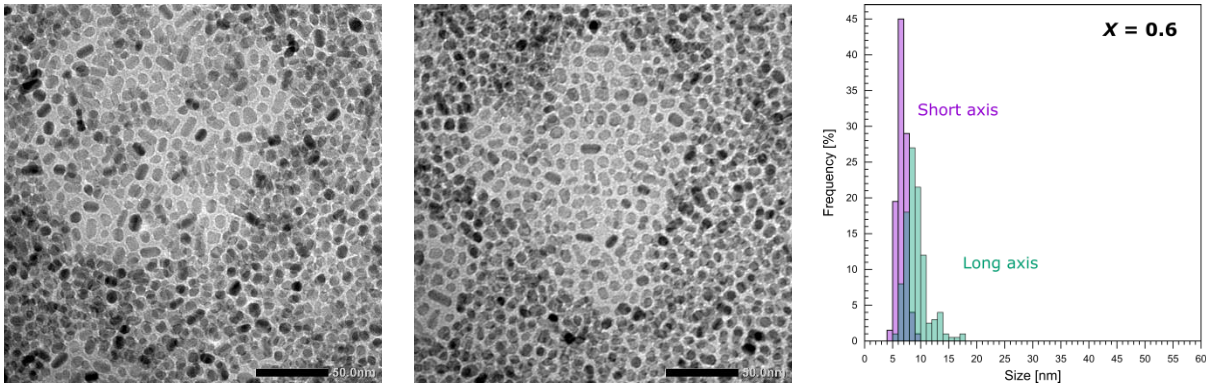

X = 0.6
Short axis
Long axis
Frequency [%]
Size [nm]

(continued)

X = 0.8

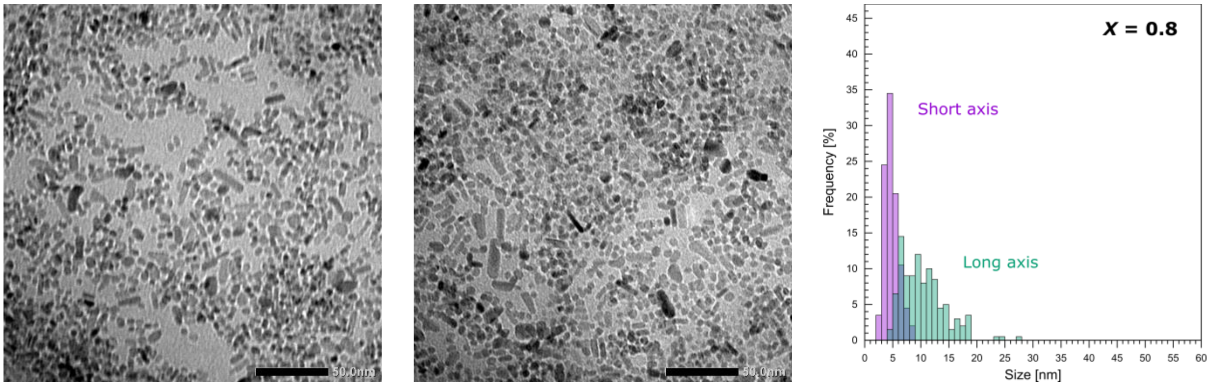

X = 1.0

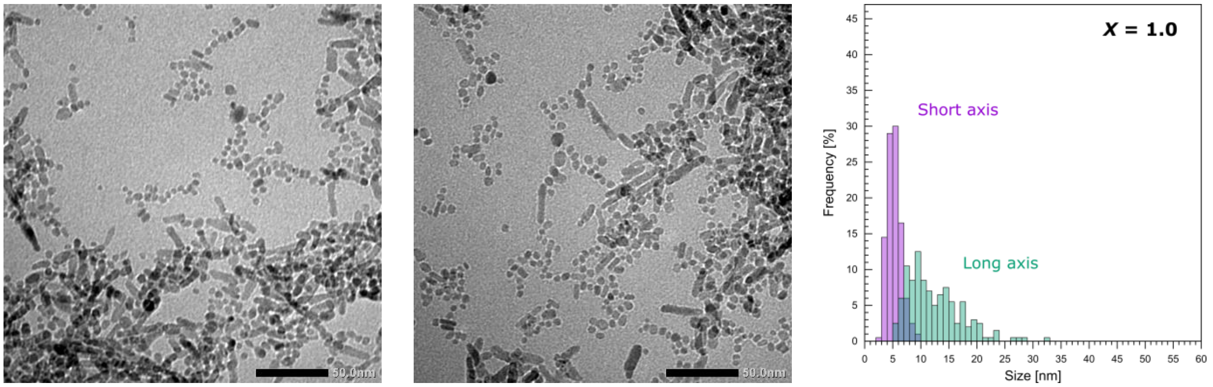

Figure S1. TEM images and size distributions of as-synthesized TiO_2 NPs. The scale bars are 50 nm.

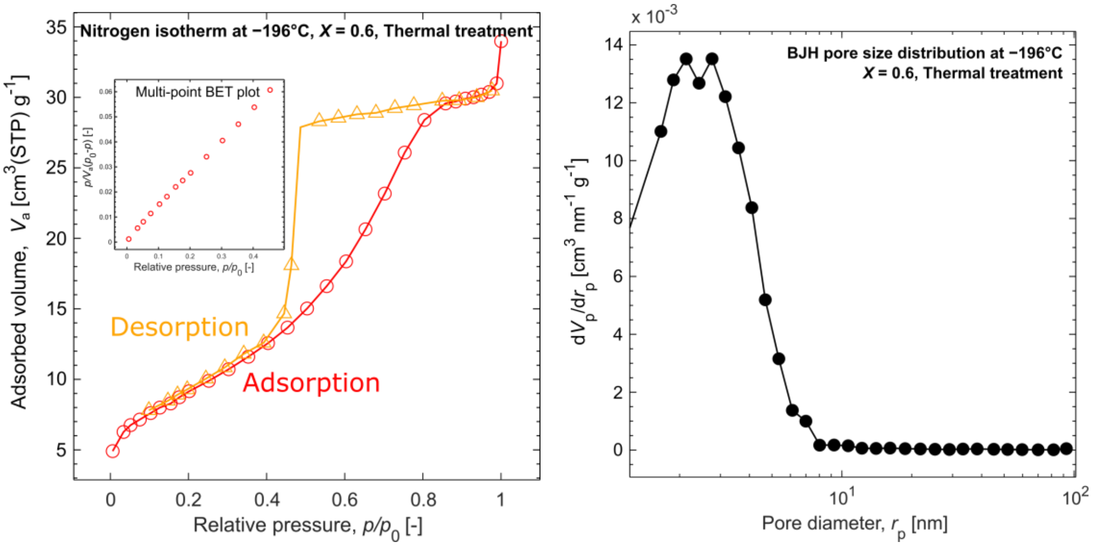

Figure S2. (left) N_2 adsorption–desorption isotherms, (inset) BET plots, and (right) Barrett-Joyner-Halenda (BJH) pore size distributions of TiO_2–OA/OAm (X = 0.6) after thermal treatment at 600 °C. The Specific surface area was obtained as 33.2 m^2/g.

$X = 0.0$

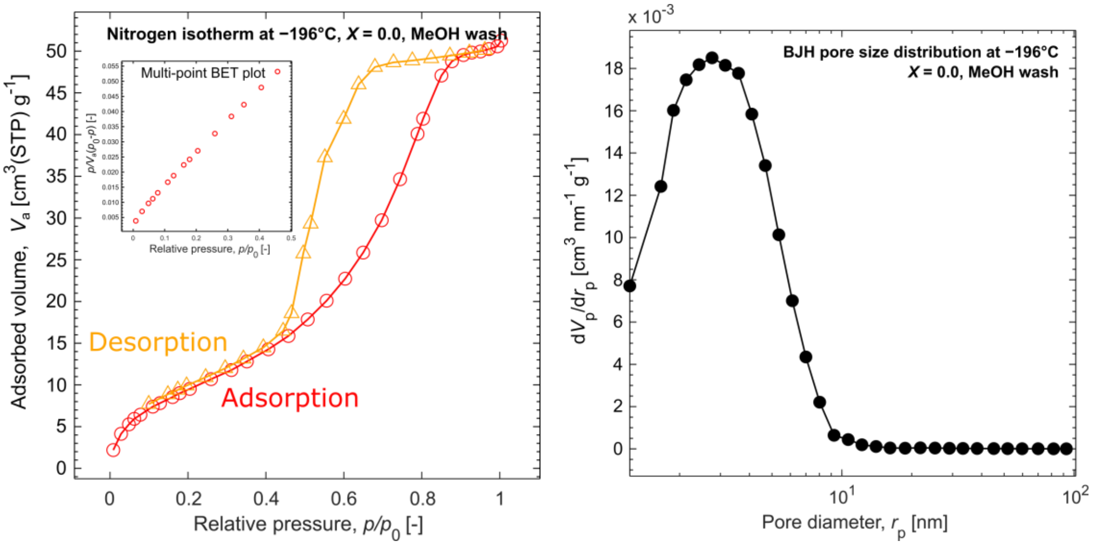

(continued)

$X = 0.3$

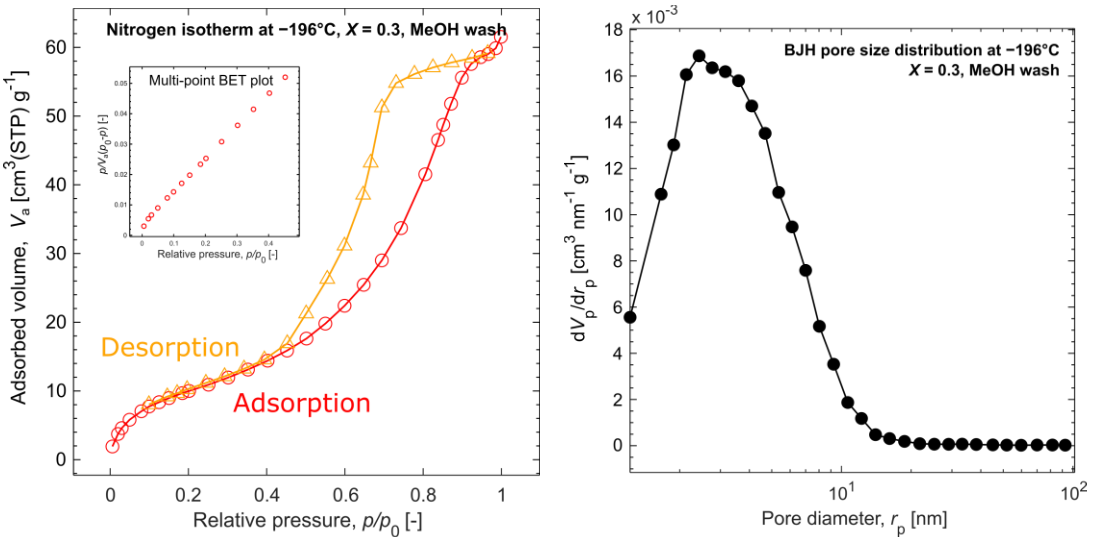

$X = 0.6$

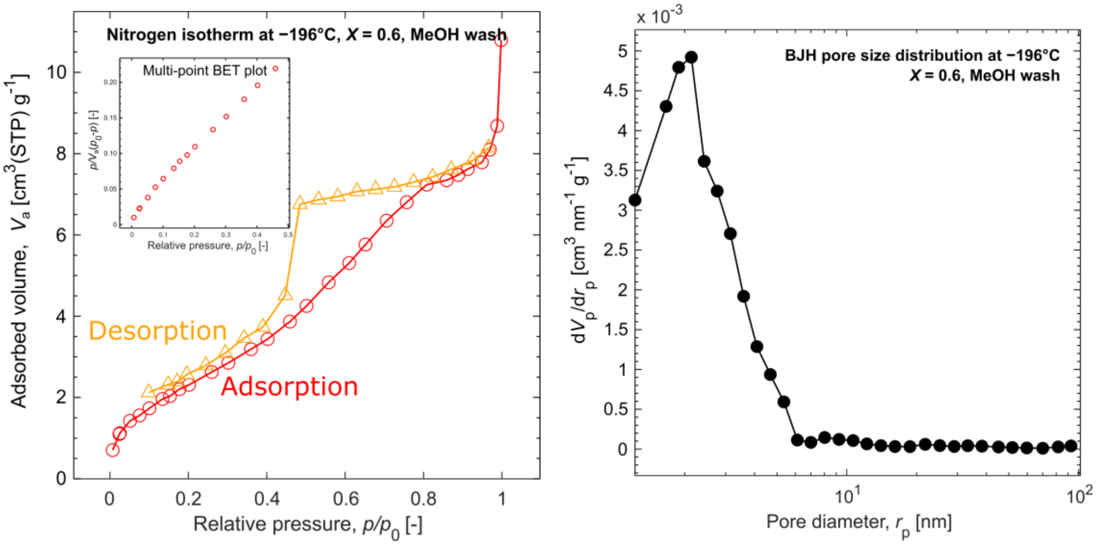

(continued)

$X = 0.8$

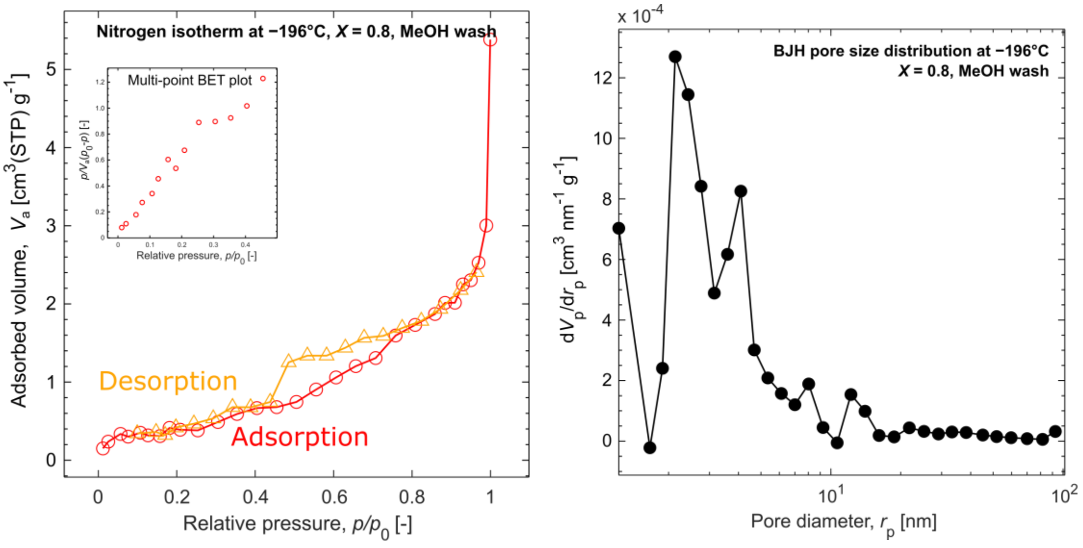

$X = 1.0$

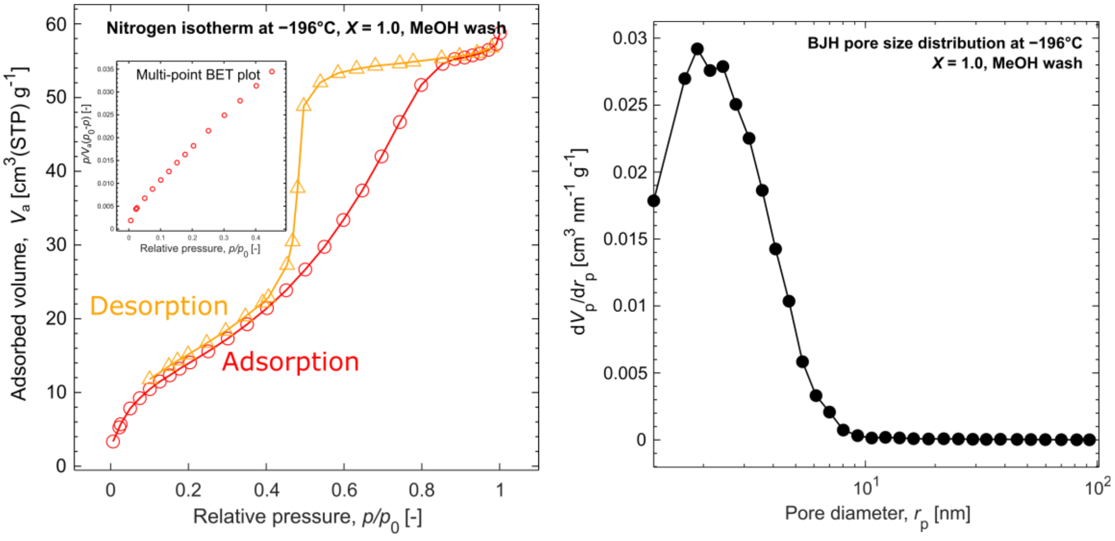

Figure S3. (left) N_2 adsorption–desorption isotherms, (inset) BET plots, and (right) BJH pore size distributions for TiO_2–OA/OAm washed with methanol.

$X = 0.0$

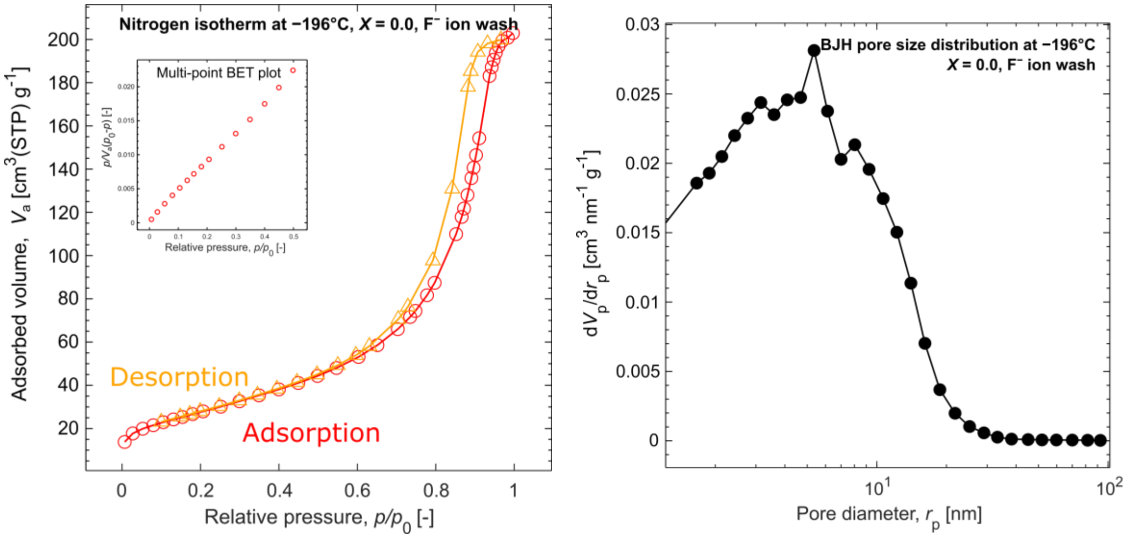

Nitrogen isotherm at −196°C, X = 0.0, F⁻ ion wash
Multi-point BET plot
Relative pressure, p/p0 [-]
Adsorbed volume, Va [cm3(STP) g-1]
Desorption
Adsorption
BJH pore size distribution at −196°C
X = 0.0, F⁻ ion wash
dVp/drp [cm3 nm-1 g-1]
Pore diameter, rp [nm]

$X = 0.3$

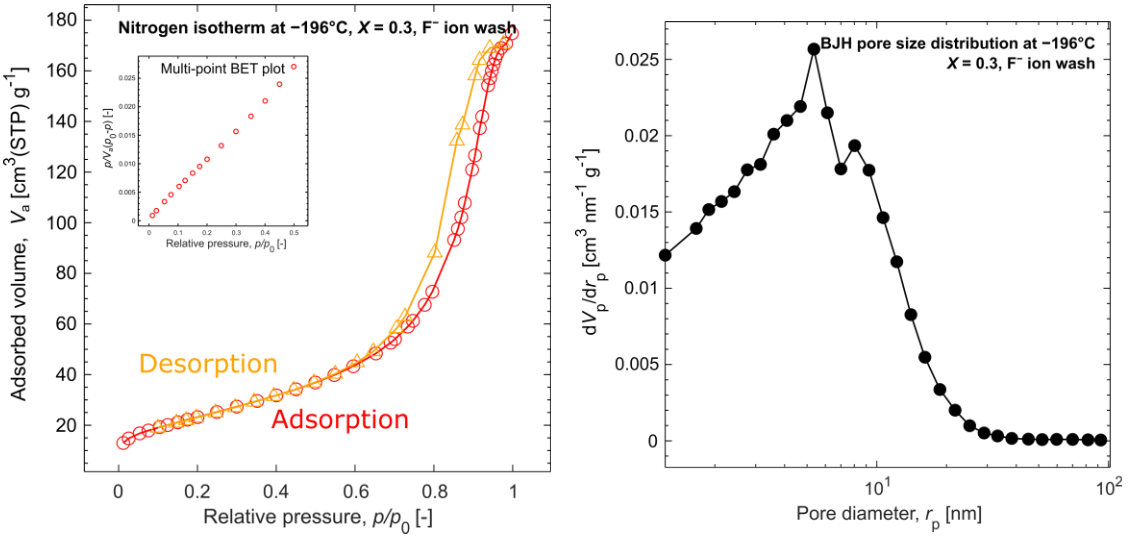

Nitrogen isotherm at −196°C, X = 0.3, F⁻ ion wash
Multi-point BET plot
Relative pressure, p/p0 [-]
Adsorbed volume, Va [cm3(STP) g-1]
Desorption
Adsorption
BJH pore size distribution at −196°C
X = 0.3, F⁻ ion wash
dVp/drp [cm3 nm-1 g-1]
Pore diameter, rp [nm]

(continued)

$X = 0.6$

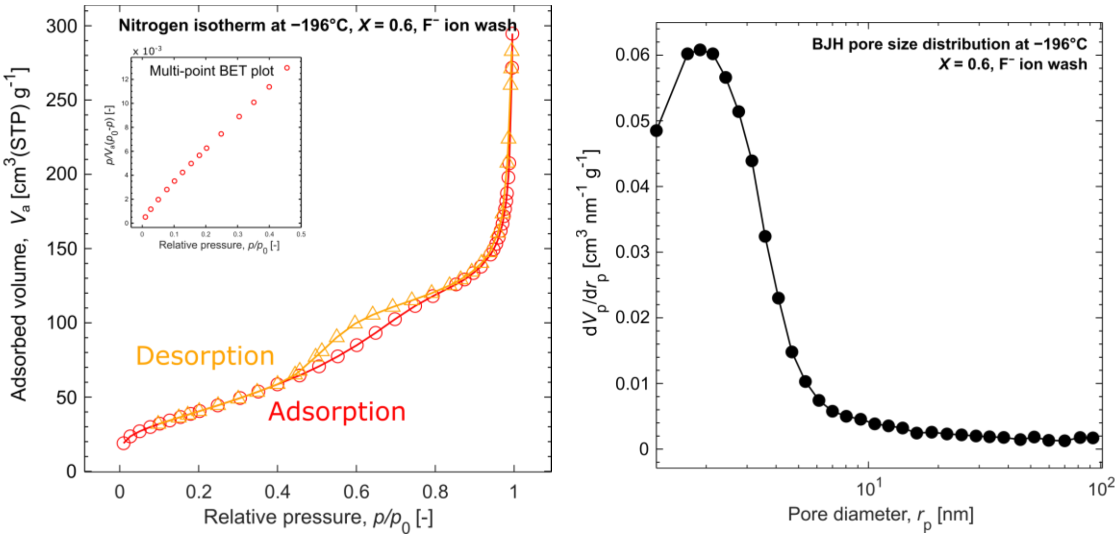

$X = 0.8$

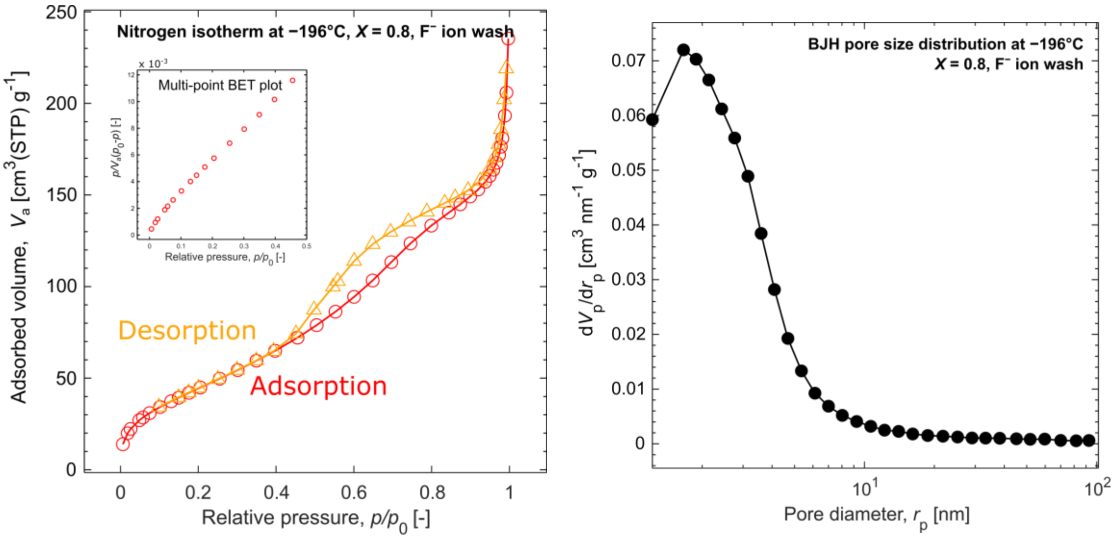

(continued)

X = 1.0

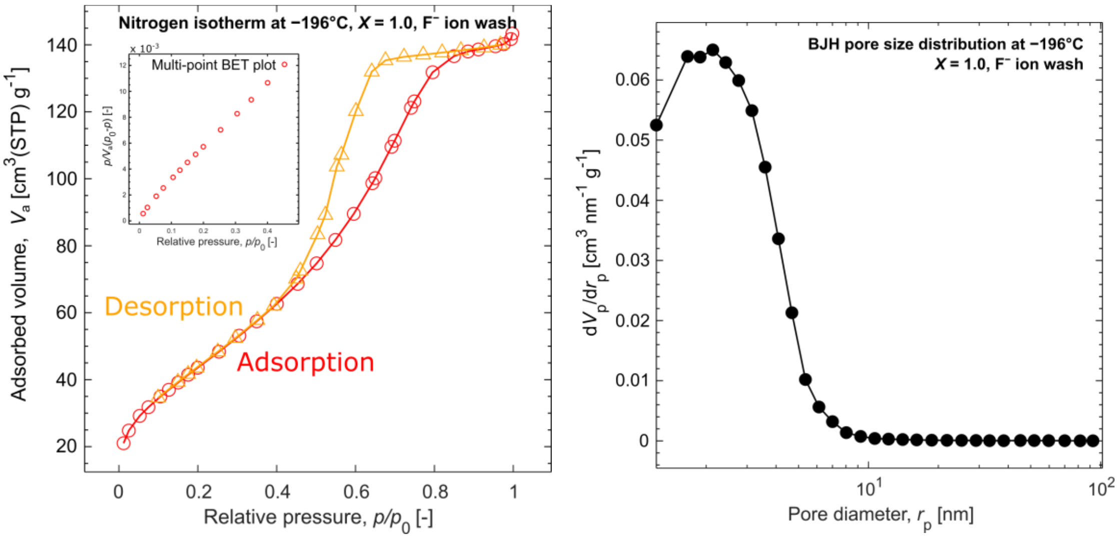

Figure S4. (left) N_2 adsorption–desorption isotherms, (inset) BET plots and (right) BJH pore size distributions for TiO_2–OA/OAm washed by F^- ions.

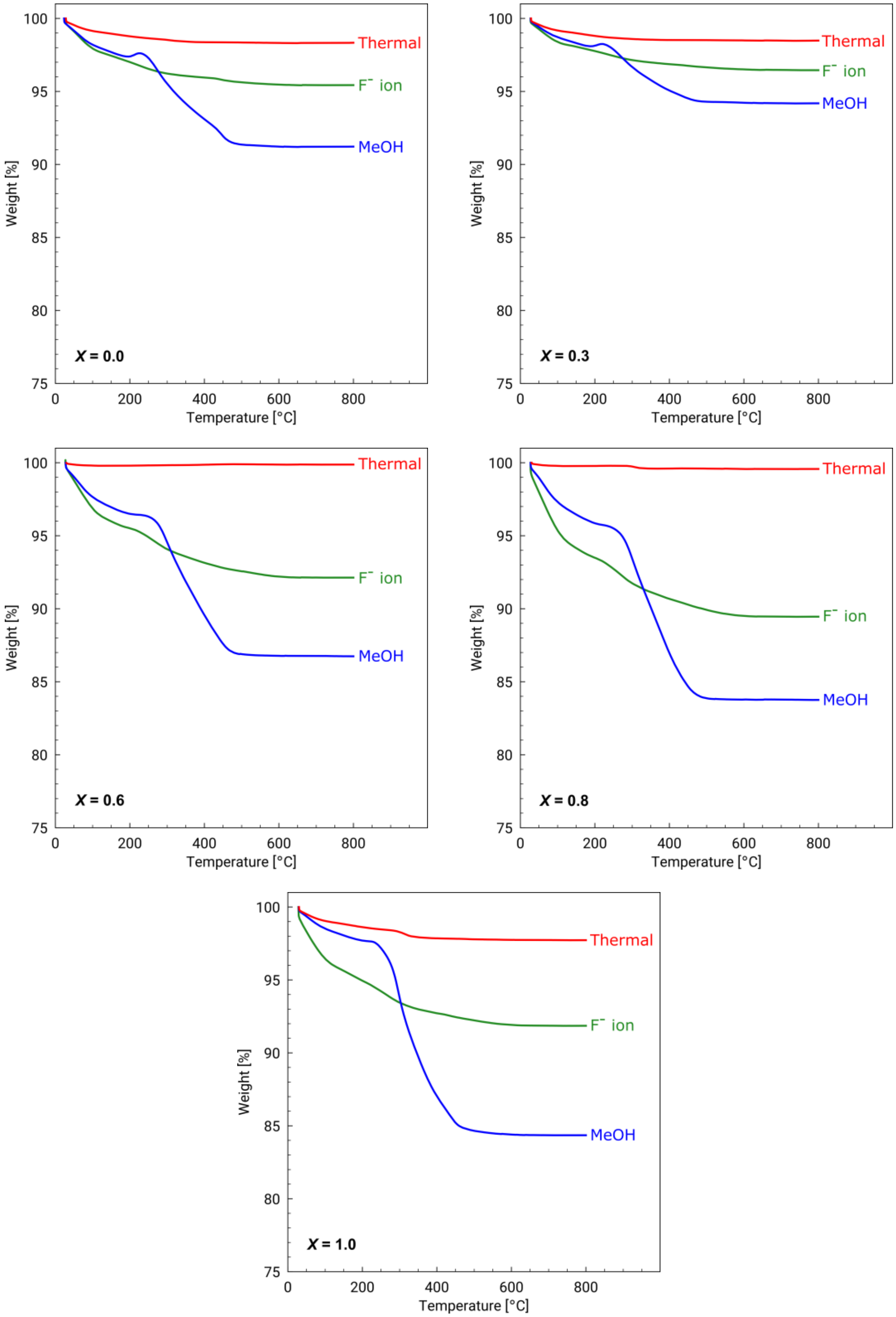

Figure S5. TGA curves of TiO_2-OA/OAm (blue) washed by methanol, (green) washed by F^- ion and (red) after thermal treatment at 600 °C.

Table S2. Calculated specific surface area estimated by TEM observations of as-synthesized TiO_2 NPs.

X = [OA]/([OA] + [OAm])	Specific surface area[a] [m^2/g]
0.0	49.8–183
0.3	42.6–183
0.6	137–261
0.8	108–427
1.0	89.4–385

[a] Values of specific surface area are calculated under the assumption of truly spherical anatase particles as $6/(\rho_{\mathrm{anatase}} d_{\mathrm{TEM}})$. The upper limit was derived from minor axes minus SD and the lower limit from major axes plus SD. We used 3.90 as ρ_{anatase} for the estimation.

$X = 0.0$

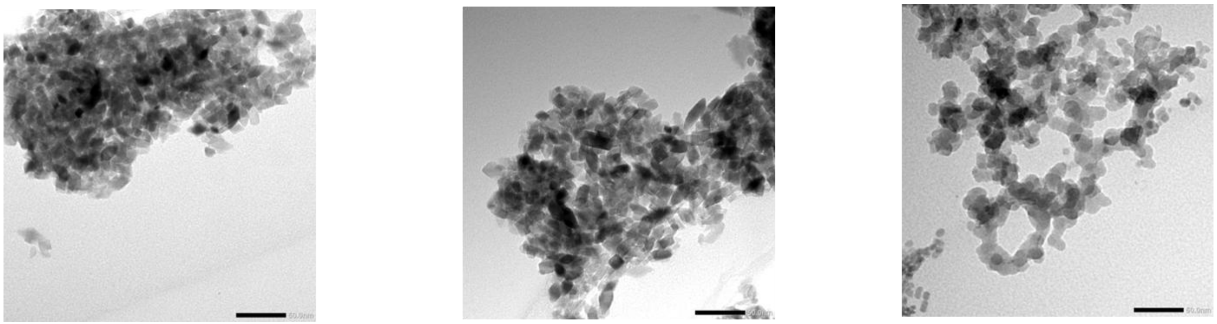

$X = 0.3$

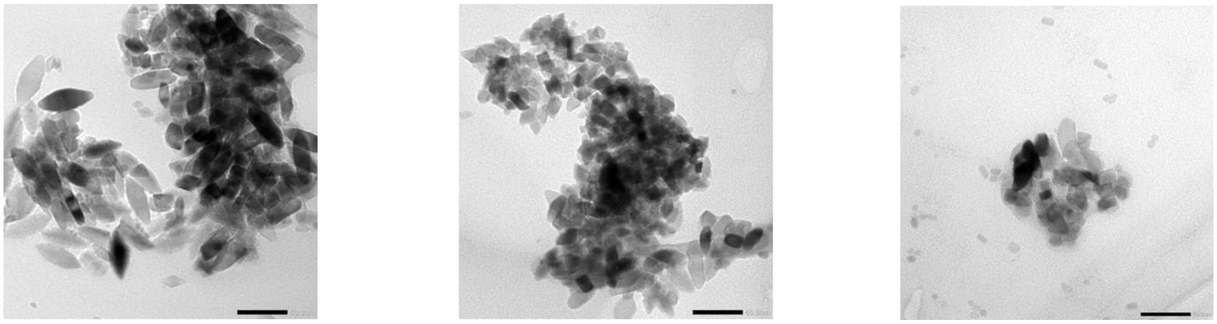

$X = 0.6$

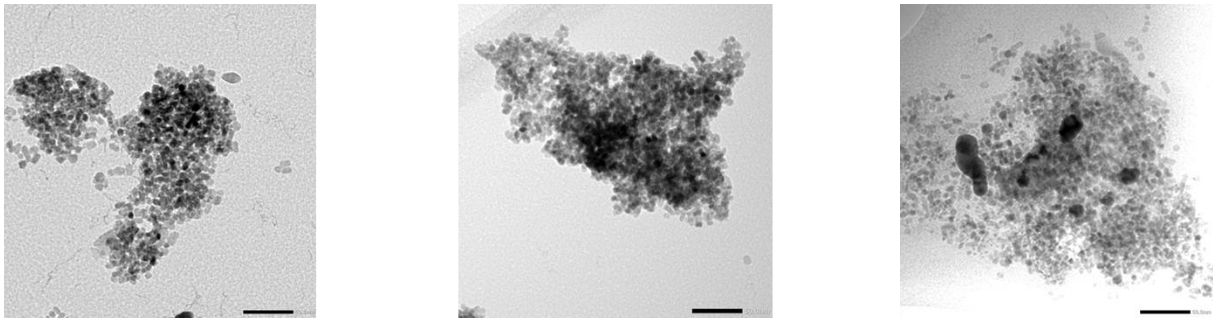

(continued)

$X = 0.8$

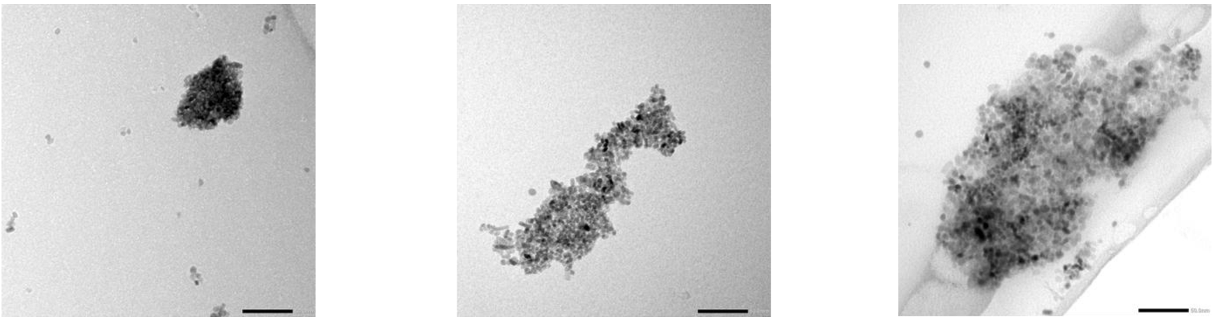

$X = 1.0$

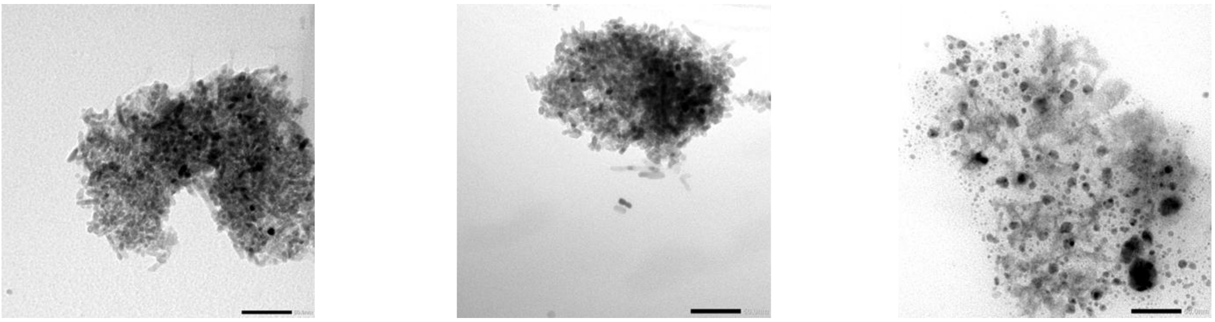

Figure S6. TEM images of TiO_2 NPs (left) washed by methanol, (center) washed by F^{-} ion, and (right) after thermal treatment. The scale bars are 50 nm.

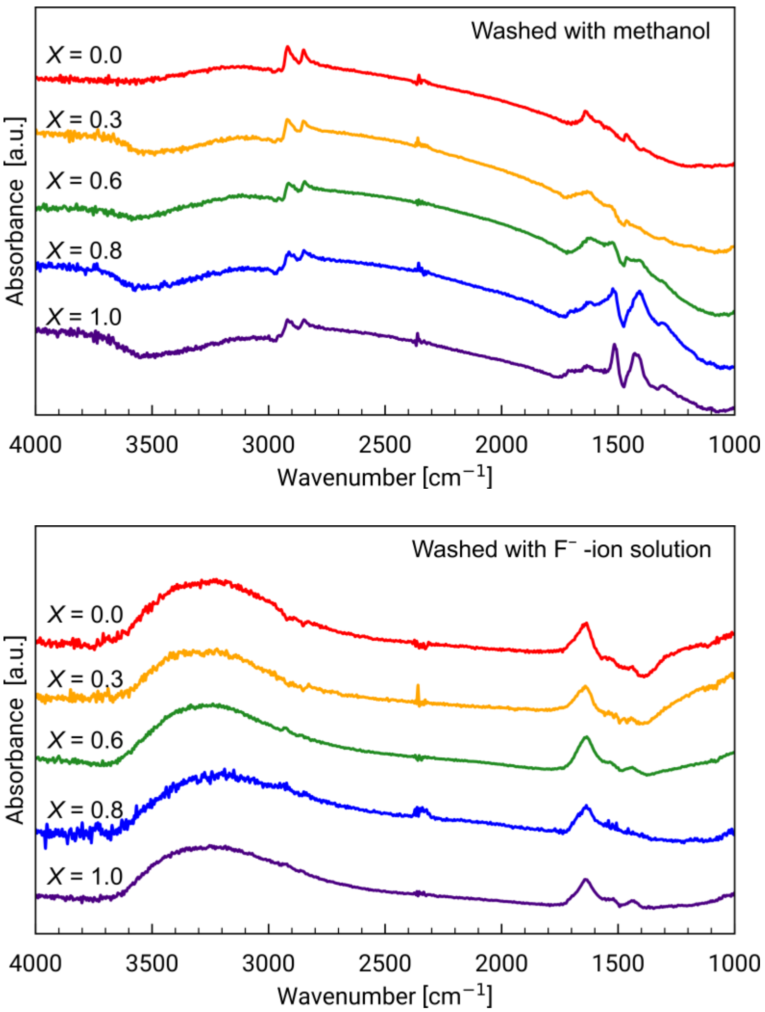

Figure S7. ATR-FTIR spectra of TiO_2 NPs washed with (above) methanol and (below) F^--ion solution.

Section S3: References

(1) Dinh, C.-T.; Nguyen, T.-D.; Kleitz, F.; Do, T.-O. Shape-Controlled Synthesis of Highly Crystalline Titania Nanocrystals. *ACS Nano* **2009**, *3* (11), 3737–3743.

(2) Shirmardi Shaghasemi, B.; Dehghani, E. S.; Benetti, E. M.; Reimhult, E. Host–Guest Driven Ligand Replacement on Monodisperse Inorganic Nanoparticles. *Nanoscale* **2017**, *9* (26), 8925–8929.
